# Association between Folate Intake and the Risk of Lung Cancer: A Dose-Response Meta-Analysis of Prospective Studies

**DOI:** 10.1371/journal.pone.0093465

**Published:** 2014-04-08

**Authors:** Yu-Fei Zhang, Li Zhou, Hong-Wei Zhang, An-Ji Hou, Hong-Fang Gao, Yu-Hao Zhou

**Affiliations:** 1 Department of Oncology, Shanghai Seventh People's Hospital, Shanghai, China; 2 Department of Rehabilitation Institute, Shanghai Seventh People's Hospital, Shanghai, China; University of North Carolina School of Medicine, United States of America

## Abstract

**Background:**

Studies have reported inconsistent results regarding the existence of an association between folate intake and the risk of lung cancer. The purpose of this study was to summarize the evidence from prospective cohort studies regarding this relationship by using a dose-response meta-analytic approach.

**Methodology and Principal Findings:**

In September 2013, we performed electronic searches in PubMed, Embase, and the Cochrane Library to identify studies examining the effect of folate intake on the incidence of lung cancer. Only prospective cohort studies that reported the effect estimates about the incidence of lung cancer with 95% confidence intervals (CIs) for more than 2 categories of folate intake were included. Overall, we examined 9 cohort studies reporting the data of 566,921 individuals. High folate intake had little effect on the risk of lung cancer (risk ratio [RR], 0.92; 95% CI, 0.84–1.01; P = 0.076). Dose-response meta-analysis also suggested that a 100 µg/day increase in folate intake had no significant effect on the risk of lung cancer (RR, 0.99; 95% CI, 0.97–1.01; P = 0.318). Subgroup analysis suggested that the potential protective effect of low folate intake (100–299 µg/day) was more evident in women than men, while the opposite was true of high folate intake (>400 µg/day). Finally, subgroup analyses of a 100 µg/day increment in folate intake indicated that its potential protective effect was more evident in men than in women.

**Conclusion/Significance:**

Our study revealed that folate intake had little or no effect on the risk of lung cancer. Subgroup analyses indicated that an increased folate intake was associated with a reduced risk of lung cancer in men. Furthermore, low folate intake may be a protective factor for women, and high folate intake for men.

## Introduction

Lung cancer is the leading cause of cancer-related deaths worldwide for both men and women, and approximately 1.5 million new cases are diagnosed each year [1]–[2]. For the past few decades, studies have shown that fruits and vegetables are associated with a lower incidence of lung cancer [3]; therefore, supplemental vitamins are widely used for chemoprevention of lung cancer because dietary habits are difficult to change [4]. Furthermore, folate is a precursor of the main coenzyme involved in the transfer of one-carbon groups essential for DNA synthesis and methylation; therefore, folate has been hypothesized to be associated with the risk of cancer [5]–[6].

Epidemiological studies have suggested that a healthy diet and lifestyle are critical for the prevention of lung cancer [7]–[8]. Dietary intake of fruits and vegetables has been closely related to the risk of lung cancer. Among the supplemental vitamin subtypes, folate shows promise for inhibiting carcinogenesis and reducing the risk of lung cancer at a certain dose. However, data on the effect of folate intake on the subsequent incidence of lung cancer are limited and inconclusive.

The results of a previous prospective study indicated that high folate intake was associated with a lower risk of lung cancer [9], whereas another study showed that it had limited effects in serum [10]. It is particularly important to clarify the optimal daily folate intake, as it has not been definitively determined. In this study, we performed a dose-response meta-analysis of available prospective studies to determine the association between folate intake and the incidence of lung cancer. We also performed a dose-response meta-analysis to quantify the risk of lung cancer with an incremental increase in folate intake in the general population.

## Methods

### Data Sources, Search Strategy, and Selection Criteria

This review was conducted and reported according to the Preferred Reporting Items for Systematic Reviews and Meta-Analysis Statement issued in 2009 (Checklist S1) [11]. Any prospective study that examined the relationship between folate intake and incidence of lung cancer was eligible for inclusion in our study, and no restrictions were placed on language or publication status (published, in press, or in progress). We searched PubMed, Embase, and the Cochrane Library electronic databases for articles published until September 2013, and used “(“folate” OR “folic acid”) AND (“cancer” OR “neoplasm” OR “carcinoma”) AND (“cohort” OR “cohort studies” OR “nest case-control studies”)” as the search terms. We also conducted manual searches of reference lists from all the relevant original and review articles to identify additional eligible studies. The medical subject heading, methods, patient population, design, exposure, and outcome variables of these articles were used to identify the relevant studies.

The literature search was independently undertaken by 2 authors (YFZ and HFG) with a standardized approach. Any inconsistencies between these 2 authors were settled by the primary author (YHZ) until a consensus was reached. The study was eligible for inclusion if the following criteria were met: (1) the study had a prospective design (prospective cohort or prospective nested case-control study); (2) the study investigated the association between folate intake and the risk of lung cancer; and (3) effect estimates (risk ratio [RR], hazard ratio [HR], or odds ratio [OR]), and 95% confidence intervals (CIs) were used to compare high and low folate intake. We excluded all case-control studies because various confounding factors could bias the results.

### Data Collection and Quality Assessment

The data collected included the first author or study group's name, publication year, country, study design, assessment of folate exposure, sample size, age at baseline, sex, follow-up duration, effect estimates and 95% CIs, comparison categories, and covariates in the fully adjusted model. We also extracted the numbers of cases/persons or person-years, effect of the different exposure categories, and 95% CIs. For studies that reported several multivariable adjusted RRs, we selected the effect estimate maximally adjusted for potential confounders.

The Newcastle-Ottawa scale (NOS) [12], which is quite comprehensive and has been partially validated for evaluating the quality of observational studies in a meta-analysis, was used to evaluate the methodological quality [13]. The NOS is based on the following 3 subscales: selection (4 items), comparability (1 item), and outcome (3 items); a “star system” (range, 0–9) is used for assessment (Table S1). The data extraction and quality assessment were independently conducted by 2 authors (HFG and LZ). Information was examined and adjudicated independently by an additional author (YHZ) after referring to the original studies.

### Statistical Analysis

We examined the relationship between folate intake and the risk of lung cancer on the basis of the effect estimates (RR, OR, or HR) and 95% CIs published in each study. First, we used the random-effects model [14]–[15] to calculate summary RRs and 95% CIs for high versus low folate intake. Second, we transformed category-specific risk estimates into RR estimates associated with an increase in folate intake of 100 µg/day by using the method of generalized least squares for trend estimation [16]. These estimates were calculated by assuming a linear relationship between the natural logarithm of RR and increasing folate intake. The value assigned to each folate category was the mid-point for closed categories and the median for open categories (assuming a normal distribution for folate intake). We combined the RRs for each 100 µg/day increase in folate intake by using the results of a random-effect meta-analysis [14]. Third, we conducted a dose-response random-effects meta-analysis from the correlated natural logarithm of RRs or HRs across the folate intake categories [16]–[17]. To derive the dose-response curve, we modeled folate by using restricted cubic splines with 3 knots at fixed percentiles, 10%, 50%, and 90%, of the distribution [16]. This method requires knowledge about the distribution of cases and persons or person-years as well as effect estimates (RRs, OR, or HRs) with the variance estimates for at least 3 quantitative exposure categories. Fourth, folate intake was also analyzed by considering a study-specific dose, and the lowest intake category was used as the reference throughout the analyses. If no participants were diagnosed with lung cancer in a study's highest intake category, the participants in the highest category were included in the second highest intake category.

Heterogeneity between studies was investigated by using the Q statistic, and we considered P-values <0.10 indicative of significant heterogeneity [18]–[19]. Subgroup analyses were conducted according to the country, sex, and duration of follow-up. In addition, we performed a sensitivity analysis by removing each individual study from the meta-analysis. Several methods were used to check for potential publication bias. Visual inspections of funnel plots for incidence of lung cancer were conducted. The Egger [20] and Begg [21] tests were also used to statistically assess the publication bias for the incidence of lung cancer. All reported P-values are 2-sided, and P-values <0.05 were considered statistically significant for all included studies. Statistical analyses were performed by using STATA software (version 12.0; Stata Corporation, College Station, TX, USA).

## Results

**Figure 1:**
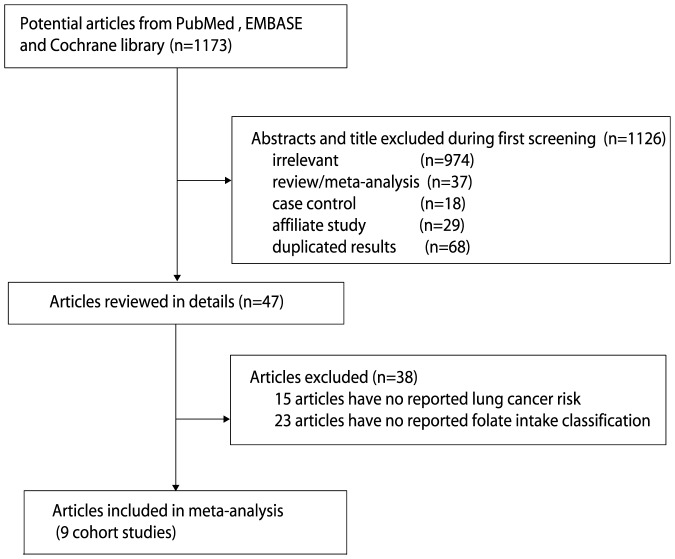
Flow diagram of the literature search and studies selection process.

The results of the study selection process are shown in Figure 1. We identified 1,173 articles in our initial electronic search; 1,126 duplicate and irrelevant studies were excluded. A total of 47 potentially eligible studies were selected. After a detailed evaluation, 9 prospective studies [9], [22]–[29] were selected for the final meta-analysis. A manual search of the reference lists of these studies did not yield any new eligible studies. The general characteristics of the included studies are presented in Table 1.

**Table 1:** Baseline characteristic of studies included in the systematic review and meta-analysis.

Study	Country	Study design	Assessment of exposure	Sample size	Men/women	Age at baseline	Effect estimate	Comparison categories	Follow-up (year)	Covariates in fully adjusted model
EV Bandera 1997 [22]	US	cohort	FFQ	48000	27544/20456	40–80	RR	Tertiles 3 vs Tertiles 1	8.0	age, education, cigarettes per day, years smoking, and total energy intake
LE Voorrips 2000 [9]	Netherland	cohort	FFQ	58279	58279/0	55–69	RR	400 vs 212 µg/d	6.3	current smoking, years of smoking cigarettes, number of cigarettes per day, highest educational level, family history of lung cancer, age, socioeconomic status, folate, energy.
JM Yuan 2003 [23]	Singapore	cohort	FFQ	63257	27959/35298	45–74	RR	quintile 5 vs quintile 1	5.3	age at baseline, sex, dialect group, year of interview, level of education, BMI, number of cigarettes smoked per day, number of years of smoking, and number of years since quitting smoking for former smokers
CG Slatore 2007 [24]	US	cohort	FFQ	77721	37872/39849	50–76	HR	>400 vs <200 µg/d	10.0	age, sex, years smoked, pack-years, and pack-years squared
GC Kabat 2008 [25]	Canada	cohort	FFQ	89835	0/89835	40–59	HR	>374 vs <237 µg/d	16.4	age, BMI, pack-years of smoking, years of education, menopausal status, family history of breast cancer, history of breast biopsy, age at menarche, parity, oral contraceptive use, hormone replacement therapy, intake of calories, and alcohol intake.
N Roswall 2010 [26]	Denmark	cohort	FFQ	55557	26489/29068	50–64	RR	>383.7 vs <247.9 µg/d	10.6	intake of the other micronutrients, further for smoking status, smoking duration, smoking intensity, possible cessation and when, passive smoking and work exposure.
JK Bassett 2012 [27]	Australia	cohort	FFQ	41514	14595/22451	40–69	HR	>80^th^ vs <20^th^	15.0	country of birth, smoking status, time since cessation of smoking, pack-years of smoking, sex, alcohol consumption, β-carotene intake, BMI, physical activity and daily energy intake.
Y Takata 2012 [28]	China	cohort	FFQ	71267	0/71267	40–70	HR	405 vs 185 µg/d	11.2	age, passive smoking, total caloric intake, income, occupation, BMI category, and history of asthma.
Y Takata 2013 [29]	China	cohort	FFQ	61491	61491/0	40–74	HR	474.4 vs 217.9 µg/d	5.5	age, yr of smoking, the number of cigarettes smoked per day, current smoking status, total caloric intake, education, BMI category, ever consumption of tea, history of chronic bronchitis, and family history of lung cancer among first-degree relatives.

The final 9 [9], [22]–[29] were prospective cohort studies comprising a total of 566,921 individuals. Between 41,514 and 89,835 individuals were included in each study, and follow-up periods were 5.3–16.4 years. Two studies were conducted in the US [22], [24], 2 in Europe [9], [26], 3 in Asia [23], [28], [29], 1 in Australia [27], and 1 in Canada [25]. The quality of a study was assessed by using NOS [12] (Table S1), and a score ≥7 indicated high quality. Overall, 6 studies had a score of 9 [22], [24]–[28], 2 had a score of 8 [23], [29], and the remaining study had a score of 7 [9].

**Figure 2:**
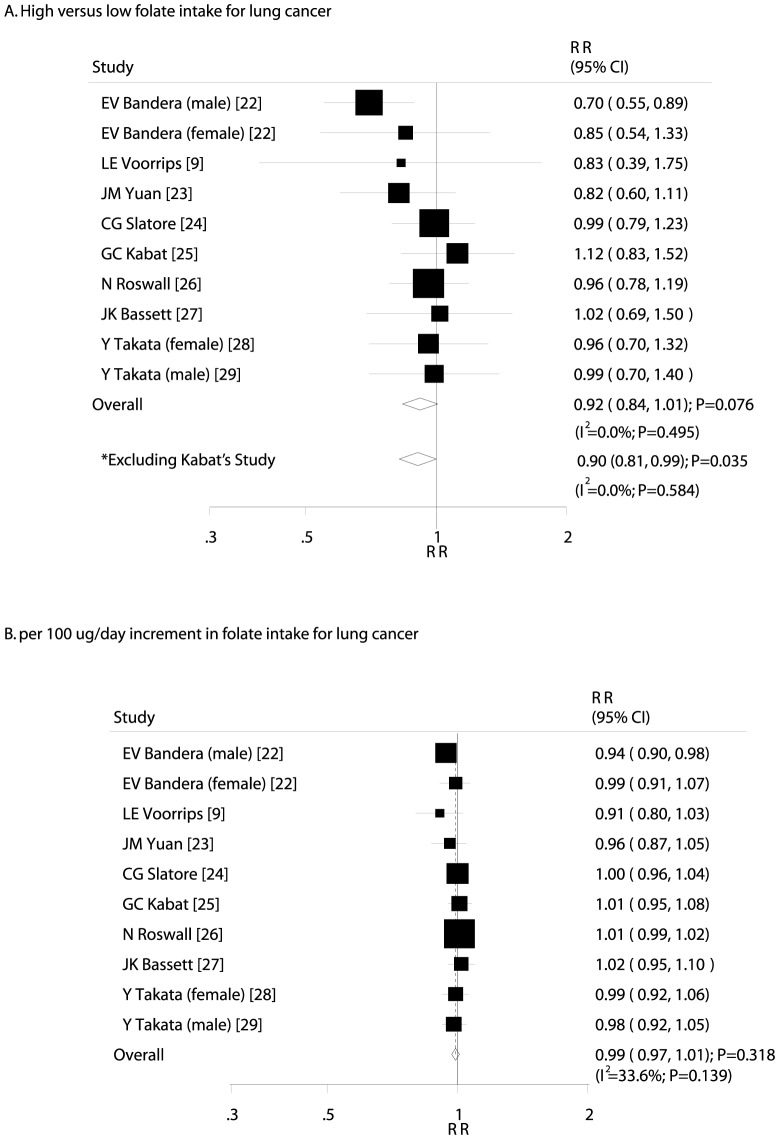
Relative risk estimates of lung cancer for high versus low folate intake (A); Dose-response meta-analysis for per 100 µg/day increment in folate intake for lung cancer (B).

After pooling these studies, the summary RR showed that a high folate intake was not associated with the incidence of lung cancer (RR, 0.92; 95% CI, 0.84–1.01; P = 0.076; Figure 2A), and no evidence of significant heterogeneity was observed (I^2^ = 0.0%; P = 0.495). Therefore, sensitivity analyses were also conducted, and after each study was sequentially excluded from the pooled analysis, we noted that a high folate intake was associated with a reduction in the risk of lung cancer when the study by Kabat et al. was excluded [25] (RR, 0.90; 95% CI, 0.81–0.99; P = 0.035; without evidence of heterogeneity; Figure 2A). This study specifically included women and had the longest follow-up periods. However, the conclusion was not affected by the exclusion of any other study. The dose-response meta-analysis findings did not suggest any association between the risk of lung cancer and a 100 µg/day increase in folate intake (RR, 0.99; 95% CI, 0.97–1.01; P = 0.318; Figure 2B), with moderate heterogeneity across studies (I^2^ = 33.6%; P = 0.139).

**Figure 3:**
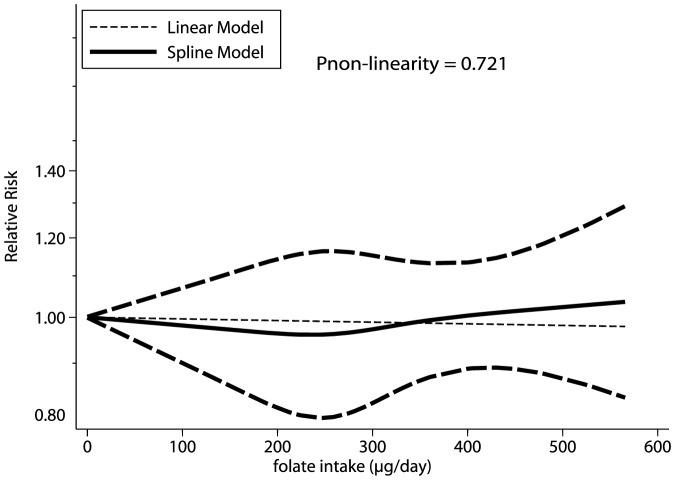
Dose-response relations between folate intake and relative risks of lung cancer.

All studies were included in the dose-response curve to determine the relationship between folate intake and the incidence of lung cancer. As shown by the P-value of nonlinearity (P = 0.721), there was no evidence of a potential nonlinear relationship (Figure 3). The effect on the risk of lung cancer of different categories of folate dose intake compared with the lowest intake category was also evaluated (Table 2). No significant differences were identified between low (100–299 µg/day), median (300–399 µg/day), and high doses (≥400 µg/day), and the lowest folate intake.

**Table 2:** Subgroup analysis of lung cancer for folate intake versus the lowest intake.

Subgroup	100–299 µg per day folate intake	P value	300–400 µg per day folate intake	P value	>400 µg per day folate intake	P value
Country						
US	1.07 (0.83–1.37)	0.597	1.05 (0.81–1.36)	0.712	0.84 (0.71–1.00)	0.051
Europe	0.98 (0.87–1.12)	0.799	0.83 (0.49–1.42)	0.501	0.95 (0.78–1.16)	0.620
Asia	0.90 (0.80–1.01)	0.070	0.95 (0.77–1.17)	0.647	0.97 (0.77–1.23)	0.822
Other	0.85 (0.68–1.07)	0.169	0.88 (0.64–1.21)	0.431	1.11 (0.91–1.35)	0.318
Sex						
Men	0.85 (0.69–1.06)	0.144	0.77 (0.53–1.11)	0.160	0.77 (0.66–0.89)	0.001
Women	0.78 (0.63–0.97)	0.023	0.86 (0.66–1.13)	0.274	1.02 (0.85–1.22)	0.835
Both	1.01 (0.96–1.06)	0.742	1.03 (0.96–1.09)	0.425	1.00 (0.88–1.15)	0.954
The duration of the follow-up period (years)						
10 or more	0.95 (0.84–1.07)	0.403	0.98 (0.88–1.10)	0.782	1.01 (0.91–1.13)	0.815
Less than 10	0.91 (0.81–1.02)	0.112	0.77 (0.53–1.11)	0.160	0.81 (0.70–0.93)	0.004
Overall	0.99 (0.94–1.04)	0.618	0.95 (0.84–1.07)	0.387	0.93 (0.84–1.02)	0.126

Heterogeneity testing for the analysis revealed P>0.10 for the incidence of lung cancer. We concluded that heterogeneity is not significant in the overall analysis, suggesting that most variation was attributable to chance alone. Subgroup analyses were conducted to evaluate the effect of folate on the risk of lung cancer in a specific population. Overall, subgroup analysis for folate intake versus the lowest intake indicated that low folate intake was associated with a reduction in the risk of lung cancer in women (RR, 0.78; 95% CI, 0.63–0.97; P = 0.023; Table 2). Furthermore, high folate intake was associated with a reduced risk of lung cancer in men (RR, 0.77; 95% CI, 0.66–0.89; P = 0.001; Table 2), or if the duration of the follow-up period was <10 years (RR, 0.81; 95% CI, 0.70–0.93; P = 0.004; Table 2). Subgroup analysis revealed that a 100 µg/day increment in folate intake may be a protective factor in men (RR, 0.95; 95% CI, 0.92–0.98; P = 0.003; Table 3), or if the follow-up duration was <10 years (RR, 0.96; 95% CI, 0.93–0.98; P = 0.003; Table 3). No other significant differences in the effects of folate intake and the risk of lung cancer were identified.

**Table 3:** Subgroup analysis for per 100 µg/day increment in folate intake for lung cancer.

Subgroup	Number of included studies	RR and 95%CI	P value	Heterogeneity (%)	P value for heterogeneity
Country					
US	2	0.97 (0.93–1.02)	0.244	55.5	0.106
Europe	2	0.98 (0.89–1.07)	0.641	61.2	0.108
Asia	3	0.98 (0.94–1.02)	0.343	0.0	0.877
Other	2	1.01 (0.97–1.06)	0.564	0.0	0.843
Sex					
Men	3*	0.95 (0.92–0.98)	0.003	0.0	0.467
Women	3*	1.00 (0.96–1.04)	0.929	0.0	0.895
Both	4	1.01 (0.99–1.02)	0.244	0.0	0.716
The duration of the follow-up period (years)					
10 or more	5	1.01 (1.00–1.02)	0.207	0.0	0.967
Less than 10	4	0.96 (0.93–0.98)	0.003	0.0	0.654

* Bandera's study reported men and women separately.

**Figure 4:**
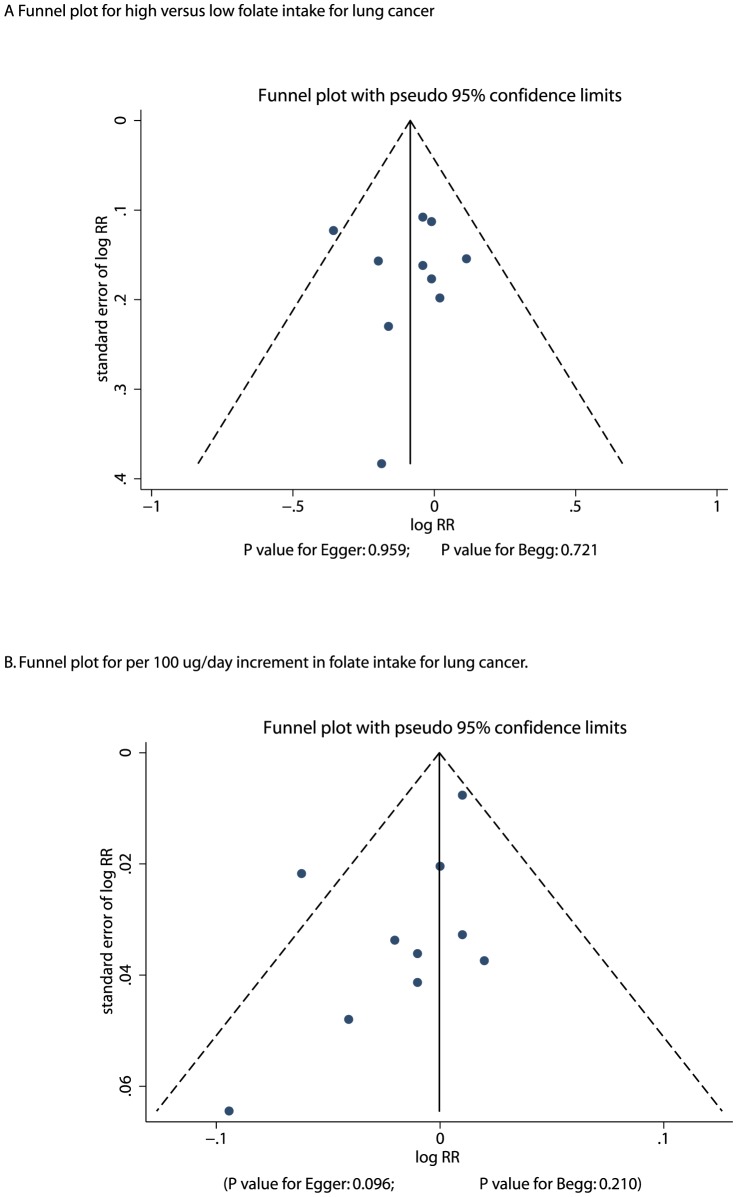
Funnel plot for high versus low folate intake for lung cancer (A); Funnel plot for per 100 µg/day increment in folate intake for lung cancer (B).

A funnel plot review could not rule out the potential for publication bias for lung cancer (Figure 4). However, the Egger [20] and Begg test [21] results did not show any evidence of publication bias, P = 0.959 and P = 0.721 for high versus low folate intake, respectively, and P = 0.096 and P = 0.210 for a 100 µg/day increment in folate intake, respectively.

## Discussion

Previous observational studies of the association between folate intake and the risk of lung cancer have been inconclusive. Three case-control studies found a decreased risk of lung cancer with high folate intake [30]–[32], and several other case-control studies [33]–[35] failed to find a significant association, although the observed ORs were below unity. Moreover, various confounding factors in case-control studies could bias the results, and the cut-off value for optimal folate intake categories differed between studies. A previous meta-analysis [36] suggested that high folate intake had no significant benefit or adverse effect on the risk of lung cancer. However, that study incorporated not only folate but also supplementation with vitamins A, C, and E to evaluate the association between supplements and the risk of lung cancer, and its conclusion may be unreliable since only 2 studies were included in such subset. In addition, the duration of follow-up was shorter than that required to show a clinical benefit, especially if event rates were lower than expected, requiring broad confidence intervals, i.e., no statistically significant differences. Therefore, we conducted a dose-response meta-analysis of prospective studies to determine the optimal folate intake dose.

Our current study was based on prospective studies, and we explored all possible correlations between folate intake and the risk of lung cancer. This large quantitative study included 566,921 individuals from 9 prospective cohort studies covering a broad population range. The findings from our current meta-analysis suggest that increased folate intake had no effect on the incidence of lung cancer. A sensitivity analysis suggested that high versus low folate intake might play a protective effect on the risk of lung cancer.

Among the 9 studies examined, the majority did not indicate any association between folate intake and the incidence of lung cancer, except 1 that reported a conflicting result [22]. Bandera et al. [22] demonstrated that there was a significant trend for increased folate intake to be associated with a lower risk of lung cancer in men (P = 0.0002), and the multivariate RR for the participants with tertile 3 folate intake was 0.70 (95% CI, 0.55–0.89) when compared to those with tertile 1 folate intake. The pooled results of our meta-analysis were consistent with most of the studies analyzed, and no evidence of an association between folate intake and the risk of lung cancer was noted; however, a sensitivity analysis indicated that high folate intake was associated with a reduced risk of lung cancer. Moreover, we discovered that all the pooled RR estimate points were less than 1.00 and had a potential trend to move to the left. Our dose-response curve revealed nonsignificant nonlinear relationships between folate intake and the risk of lung cancer. Therefore, we suggest that there might be a potential protective effect of high folate intake on the incidence of lung cancer; however, this protective effect may not be clinically significant and should be validated by performing further studies.

Subgroup analyses indicated that the protective effect of low folate intake was more evident in women than in men; conversely, the effect of high folate intake on lung cancer was more beneficial in men than in women. Furthermore, we also noted that a 100 µg/day increment in folate intake was associated with a reduced risk of lung cancer in men. One possible explanation for this could be higher smoking rates among men than among women. Consequently, the protective effect of folate on the risk of lung cancer may be negated by smoking among men, as it was noted that the proportion of current smokers was highest in the lowest folate intake category. Finally, it is possible that insufficient adjustment was made for smoking in several studies. In never-smokers, Bandera et al. [22] found a significant inverse association between folate intake and the risk of lung cancer in men, but not in women. Therefore, low folate intake might have a protective effect in women, while a high folate intake is required in men. Furthermore, we observed that the protective effect of increased folate intake was more evident in studies with follow-up periods of <10 years when compared to those with longer follow-up periods. This difference may be due to chance, as fewer studies were included in this subset resulting in less variation in the conclusions. Therefore, we generated a relative result and provided a comprehensive review of the model.

Three strengths of our study should be highlighted. First, only prospective studies were included, which should eliminate selection and recall bias. Second, the large sample size allowed us to quantitatively assess the association of folate intake with the risk of lung cancer, thus making it more powerful than any individual study. Third, the dose-response analysis included a wide range of folate intake, which allowed for accurate assessment of the dose relationship between folate intake and the risk of lung cancer.

Our meta-analysis has several potential limitations: (1) publication bias is very possible in the meta-analyses of published studies; (2) we could not differentiate effects of folate intake by lung cancer type, as the data were not available; and (3) the analysis used pooled data (individual data were not available), which restricted us from performing a more detailed relevant analysis and obtaining more comprehensive results.

In conclusion, our study suggested that folate intake might play an important role in the risk of developing lung cancer. Furthermore, low folate intake was associated with a reduced risk of lung cancer in women, and high folate intake provided a potential protective effect in men. Finally, increased folate intake may play an important role in the prevention of lung cancer in men. Future studies should: (1) focus on specific subpopulations to evaluate strategies for primary prevention of lung cancer, and (2) ascertain the specific type of lung cancer and analyze the effects according to the type.

## Supporting Information

Table S1 **Quality scores of prospective cohort studies using Newcastle-Ottawa Scale.**
(DOC)

Checklist S1 **PRISMA Checklist.**
(DOC)

## References

[1] Jemal A, Siegel R, Ward E, Hao Y, Xu J, et al (2009) Cancer statistics, 2009. CA Cancer J Clin 59: 225–249. 19474385 10.3322/caac.20006

[2] Wingo PA, Cardinez CJ, Landis SH, Greenlee RT, Ries LA (2003) Long-term trends in cancer mortality in the United States, 1930–1998. Cancer 97: 3133–3275. 12784323 10.1002/cncr.11380

[3] Smith-Warner SA, Spiegelman D, Yaun SS, Albanes D, Beeson WL, et al (2003) Fruits, vegetables and lung cancer: a pooled analysis of cohort studies. Int J Cancer 107: 1001–1011. 14601062 10.1002/ijc.11490

[4] Li R, Serdula M, Bland S, Mokdad A, Bowman B, et al (2000) Trends in fruit and vegetable consumption among adults in 16 US states: behavioral risk factor surveillance system, 1990–1996. Am J Public Health 90: 777–781. 10800429 10.2105/ajph.90.5.777 PMC1446230

[5] Bailey LB, Gregory JF 3rd (1999) Folate metabolism and requirements. J Nutr 129: 779–82. 10203550 10.1093/jn/129.4.779

[6] Selhub J, Miller JW (1991) The pathogenesis of homocysteinemia: interruption of the coordinate regulation by *S*-adenosylmethionine of the remethylation and transsulfuration of homocysteine. Am J Clin Nutr 55: 131–8. 10.1093/ajcn/55.1.131 1728812

[7] Riboli E, Norat T (2003) Epidemiologic evidence of the protective effect of fruit and vegetables on cancer risk. Am J Clin Nutr 78: 559S–69S. 12936950 10.1093/ajcn/78.3.559S

[8] World Cancer Research Fund/American Institute for Cancer Research (2007) Food, Nutrition, Physical Activity, and the Prevention of Cancer: A Global Perspective, Washington, DC: American Institute for Cancer Research.

[9] Voorrips LE, Goldbohm RA, Brants HAM, van Poppel GA, Sturmans F, et al (2000) A Prospective Cohort Study on Antioxidant and Folate Intake and Male Lung Cancer Risk. Cancer Epidemiol Biomarkers Prev 9: 357–365. 10794479

[10] Hartman TJ, Woodson K, Stolzenberg-Solomon R, Virtamo J, Selhub J, et al (2001) Association of the B-Vitamins Pyridoxal 5′-Phosphate (B6), B12, and Folate with Lung Cancer Risk in Older Men. Am J Epidemiol 153: 688–94. 11282797 10.1093/aje/153.7.688

[11] Moher D, Liberati A, Tetzlaff J, Altman DG (2009) PRISMA Group (2009) Preferred Reporting Items for Systematic Reviews and Meta-Analyses: The PRISMA Statement. Plos Medicine 6: e1000097. 19621072 10.1371/journal.pmed.1000097 PMC2707599

[12] Wells G, Shea B, O'Connell D (2009) The Newcastle-Ottawa Scale (NOS) for assessing the quality of nonrandomised studies in meta-analyses. Ottawa (ON): Ottawa Hospital Research Institute. Available:http://www.ohri.ca/programs/clinical_epidemiology/oxford.htm.

[13] Higgins JP, Green S (2011) Cochrane Handbook for Systematic Reviews of Interventions, Version 5.1.0. Available: www.cochrane-handbook.org.

[14] DerSimonian R, Laird N (1986) Meta-analysis in clinical trials. Control Clin Trials 7: 177–88. 3802833 10.1016/0197-2456(86)90046-2

[15] Ades AE, Lu G, Higgins JP (2005) The interpretation of random-effects metaanalysis in decision models. Med Decis Making 25: 646–54. 16282215 10.1177/0272989X05282643

[16] Orsini N, Bellocco R (2006) Generalized least squares for trend estimation of summarized dose-response data. Stata J 2006 6: 40–57.

[17] Greenland S, Longnecker MP (1992) Methods for trend estimation from summarized dose-response data, with applications to meta-analysis. Am J Epidemiol 135: 1301–09. 1626547 10.1093/oxfordjournals.aje.a116237

[18] Deeks JJ, Higgins JPT, Altman DG (2008) Analyzing data and undertaking meta-analyses. In: Higgins J, Green S, eds. Cochrane Handbook for Systematic Reviews of Interventions 5.0.1. Oxford, UK: The Cochrane Collaboration: chap 9.

[19] Higgins JPT, Thompson SG, Deeks JJ, Altman DG (2003) Measuring inconsistency in meta-analyses. BMJ 327: 557–60. 12958120 10.1136/bmj.327.7414.557 PMC192859

[20] Egger M, Davey Smith G, Schneider M, Minder C (1997) Bias in meta-analysis detected by a simple, graphical test. BMJ 315: 629–34. 9310563 10.1136/bmj.315.7109.629 PMC2127453

[21] Begg CB, Mazumdar M (1994) Operating characteristics of a rank correlation test for publication bias. Biometrics 50: 1088–1101. 7786990

[22] Bandera EV, Freudenheim JL, Marshall JR, Zielezny M, Priore RL, et al (1997) Diet and alcohol consumption and lung cancer risk in the New York State Cohort (United States). Cancer Causes and Control 8: 828–840. 9427425 10.1023/a:1018456127018

[23] Yuan JM, Stram DO, Arakawa K, Lee HP, Yu MC (2003) Dietary Cryptoxanthin and Reduced Risk of Lung Cancer: The Singapore Chinese Health Study. Cancer Epidemiol Biomarkers Prev 12: 890–898. 14504200

[24] Slatore CG, Littman AJ, Au DH, Satia JA, White E (2008) Long-Term Use of Supplemental Multivitamins, Vitamin C, Vitamin E, and Folate Does Not Reduce the Risk of Lung Cancer. Am J Respir Crit Care Med 177: 524–530. 17989343 10.1164/rccm.200709-1398OC PMC2258445

[25] Kabat GC, Miller AB, Jain M, Rohan TE (2008) Dietary intake of selected B vitamins in relation to risk of major cancers in women. Br J Cancer 99: 816–821. 18665162 10.1038/sj.bjc.6604540 PMC2528139

[26] Roswall N, Olsen A, Christensen J, Dragsted LO, Overvad K, et al (2010) Source-specific effects of micronutrients in lung cancer prevention. Lung Cancer 67: 275–281. 20004999 10.1016/j.lungcan.2009.11.010

[27] Bassett JK, Hodge AM, English DR, Baglietto L, Hopper JL, et al (2012) Dietary intake of B vitamins and methionine and risk of lung cancer. Eur J Clin Nutr 66: 182–187. 21878960 10.1038/ejcn.2011.157

[28] Takata Y, Cai Q, Beeghly-Fadiel A, Li H, Shrubsole MJ, et al (2012) Dietary B vitamin and methionine intakes and lung cancer risk among female never smokers in China. Cancer Causes Control 23: 1965–75. 23065072 10.1007/s10552-012-0074-z PMC3518409

[29] Takata Y, Xiang YB, Yang G, Li H, Gao J, et al (2013) Intakes of Fruits, Vegetables, and Related Vitamins and Lung Cancer Risk: Results from the Shanghai Men's Health Study (2002–2009). Nutr Cancer 65: 51–61. 23368913 10.1080/01635581.2013.741757 PMC3787870

[30] Shen H, Wei Q, Pillow PC, Amos CI, Hong WK, et al (2003) Dietary folate intake and lung cancer risk in former smokers: a case-control analysis. Cancer Epidemiol Biomarkers Prev 12: 980–986. 14578132

[31] Shi Q, Zhang Z, Neumann AS, Li G, Spitz MR, et al (2005) Case-control analysis of thymidylate synthase polymorphisms and risk of lung cancer. Carcinogenesis 26: 649–656. 15579479 10.1093/carcin/bgh351

[32] Hung RJ, Hashibe M, McKay J, Gaborieau V, Szeszenia-Dabrowska N, et al (2007) Folate-related genes and the risk of tobacco-related cancers in Central Europe. Carcinogenesis 28: 1334–1340. 17389614 10.1093/carcin/bgm067

[33] Le Marchand L, Yoshizawa CN, Kolonel LN, Hankin JH, Goodman MT (1989) Vegetable consumption and lung cancer risk: a population-based case-control study in Hawaii. J Natl Cancer Inst 81: 1158–1164. 2545891 10.1093/jnci/81.15.1158

[34] Suzuki T, Matsuo K, Hiraki A, Saito T, Sato S, et al (2007) Impact of one-carbon metabolism-related gene polymorphisms on risk of lung cancer in Japan: a case control study. Carcinogenesis 28: 1718–1725. 17468511 10.1093/carcin/bgm104

[35] Vaissiere T, Hung RJ, Zaridze D, Moukeria A, Cuenin C, et al (2009) Quantitative analysis of DNA methylation profiles in lung cancer identifies aberrant DNA methylation of specific genes and its association with gender and cancer risk factors. Cancer Res 69: 243–252. 19118009 10.1158/0008-5472.CAN-08-2489 PMC2613548

[36] Cho E, Hunter DJ, Spiegelman D, Albanes D, Beeson WL, et al (2006) Intakes of vitamins A, C and E and folate and multivitamins and lung cancer: A pooled analysis of 8 prospective studies. Int J Cancer 118: 970–978. 16152626 10.1002/ijc.21441

