# Supplementary material for: Association between Folate Intake and the Risk of Lung Cancer: A Dose-Response Meta-Analysis of Prospective Studies
Source: PLoS One. 2014 Apr 8;9(4):e93465. doi: 10.1371/journal.pone.0093465 (PMC3979671; doi:10.1371/journal.pone.0093465)
Supplement: Table S1 — Quality scores of prospective cohort studies using Newcastle-Ottawa Scale. (DOC) [file pone.0093465.s001.doc]

Table S1. Quality scores of prospective cohort studies using Newcastle-Ottawa Scale.

| Study | Selection | | | | Comparability | Outcome | | | NOS |
| --- | --- | --- | --- | --- | --- | --- | --- | --- | --- |
|  | Representativeness of the exposed cohort | Selection of the non exposed cohort | Ascertainment  of folate intake | Demonstration that outcomes was not present at start of study | Comparability on the basis of the design or analysis | Assessment of outcome | Adequate follow-up duration | Adequate follow-up rate | Overall score |
| EV Bandera 1997 [22] | 1 | 1 | 1 | 1 | 2 | 1 | 1 | 1 | 9 |
| LE Voorrips 2000 [9] | 1 | 1 | 1 | 0 | 2 | 1 | 0 | 1 | 7 |
| JM Yuan 2003 [23] | 1 | 1 | 1 | 1 | 2 | 1 | 0 | 1 | 8 |
| CG Slatore 2007 [24] | 1 | 1 | 1 | 1 | 2 | 1 | 1 | 1 | 9 |
| GC Kabat 2008 [25] | 1 | 1 | 1 | 1 | 2 | 1 | 1 | 1 | 9 |
| N Roswall 2010 [26] | 1 | 1 | 1 | 1 | 2 | 1 | 1 | 1 | 9 |
| JK Bassett 2012 [27] | 1 | 1 | 1 | 1 | 2 | 1 | 1 | 1 | 9 |
| Y Takata 2012 [28] | 1 | 1 | 1 | 1 | 2 | 1 | 1 | 1 | 9 |
| Y Takata 2013 [29] | 1 | 1 | 1 | 1 | 2 | 1 | 0 | 1 | 8 |
